# Gray matter volume correlates of resilience: a coordinate-based meta-analysis

**DOI:** 10.3389/fpsyg.2026.1691912

**Published:** 2026-03-24

**Authors:** Niki Hosseini-Kamkar, Nellie Kamkar, Rupa Kalahasthi, Jihane Ghorayeb, Aamna Razaan, Annapurna Nair, Andrew A. Nicholson, Maaham Maswood

**Affiliations:** 1 Rochester Institute of Technology, Dubai, United Arab Emirates; 2 London Health Sciences Centre Research Institute, London, ON, Canada; 3 Atlas Institute for Veterans and Families, Ottawa, ON, Canada; 4 School of Psychology, University of Ottawa, Ottawa, ON, Canada; 5 The Institute of Mental Health Research, Royal Ottawa Hospital, University of Ottawa, Ottawa, ON, Canada

**Keywords:** resilience, meta-analysis, gray matter volume, MKDA, coordinate-based meta-analysis

## Abstract

**Introduction:**

Psychological resilience refers to the capacity to maintain or regain functioning in the face of adversity, yet its structural substrates remain poorly characterized.

**Methods:**

We conducted a coordinate-based meta-analysis of whole-brain voxel-based morphometry studies to identify gray-matter markers of resilience. Eleven eligible studies (*n* = 2,993) were synthesized using MKDA.

**Results:**

Across studies reporting increased gray matter volume, a cluster showed convergence on the medial frontal wall, extending from the superior and medial frontal gyrus into the dorsal cingulate, *k* = 11 studies, family-wise error–corrected at *p* < 0.05 (10,000 permutations).

**Discussion:**

The findings implicate medial frontal–cingulate architecture as a convergent correlate of resilience, consistent with models of fronto-limbic regulatory control. Despite the modest study base and variability in definitions of resilience across studies, this meta-analysis identifies the medial frontal–cingulate hub as a reproducible structural marker of resilience, offering a concrete target for translational and interventional research.

## Introduction

Psychological resilience is generally understood as the ability to maintain or quickly regain healthy psychological functioning in the face of adversity (Luthar and Cicchetti, 2000; Southwick et al., 2014). Some researchers conceptualize resilience as the maintenance of stable, healthy functioning despite significant stressors, reflected in minimal disruption or an absence of psychopathology (Bonanno and Diminich, 2013; Kobasa, 1979). In contrast, others emphasize that resilience involves a dynamic process of recovery or even growth after adversity, rather than mere invulnerability (Luthar and Cicchetti, 2000). From this perspective, a person might initially show some negative impact but subsequently recover and perhaps exceed their prior baseline – an idea closely related to concepts of post-traumatic growth (Tedeschi and Calhoun, 2004). Contrasting definitions of resilience, as either enduring stability or rebound and positive transformation, have prompted ongoing debate (Infurna and Luthar, 2017). For the present purposes, resilience is broadly considered as an outcome of preserved or quickly restored mental health after hardship, acknowledging that it may encompass both resistance to psychopathology *and* adaptive recovery processes.

Psychiatric nosology defines potentially traumatic exposure as “contact with actual or threatened death, serious injury, or sexual violence”—whether directly experienced, witnessed, learned about as occurring to a close other, or encountered through repeated or extreme exposure to aversive details in professional duties (American Psychiatric Association, 2013). At first glance, epidemiology might suggest that resilience is the model outcome after trauma exposure. In cross-national surveys, lifetime post-traumatic stress disorder (PTSD) is estimated at ~5–20% among trauma-exposed individuals, implying that only a minority of exposed persons develop PTSD and that most individuals are remarkably resilient (Kessler et al., 2017; Koenen et al., 2017).

However, apparent “high resilience” rates are sensitive to how resilience is defined and operationalized. In particular, estimates can be inflated by methodological artifacts in longitudinal analyses (Infurna and Luthar, 2016, 2018). Many foundational studies have used growth mixture modeling (GMM), an advanced longitudinal statistical technique that identifies distinct trajectories of psychological outcomes following adversity (e.g., resilience, recovery, chronic distress, or delayed distress; Infurna and Luthar, 2018). Despite these strengths, resilience research with GMM is frequently characterized by restrictive assumptions (e.g., homogeneous variances; slopes fixed at zero), and relaxing those assumptions markedly lowers resilience estimates (Infurna and Luthar, 2016, 2018). Moreover, much of the adult literature relies on single outcomes (e.g., symptoms alone), which can mischaracterize resilience. For example, when resilience is inferred solely from low PTSD or depressive symptoms, individuals who still show decrements in other domains, such as life satisfaction, positive affect, physical functioning, or general health, may nonetheless be classified as resilient, which inflates prevalence estimates (Infurna and Luthar, 2018). Consistent with this concern, after spousal loss, estimates of resilience varied sharply by domain (66% for life satisfaction vs. 19% for negative affect), and only 8% of individuals were considered resilient across all five outcomes considered jointly (Infurna and Luthar, 2017). These discrepancies underscore that resilience is multidimensional and that valid assessment requires multiple domains and repeated measurements rather than a single symptom endpoint (Infurna and Jayawickreme, 2019; Infurna and Luthar, 2018). In addition, overreliance on retrospective self-reports of “perceived growth” can inflate estimates of positive change (Infurna and Jayawickreme, 2019). Overall, operational and analytic decisions determine who is labeled “resilient,” the estimated prevalence of resilience, and the kinds of statistical models used to characterize longitudinal trajectories of psychological functioning following adversity.

Notwithstanding the caveats about how resilience is operationalized and measured, exposure to potentially traumatic events remains widespread. According to criterion A of the Diagnostic and Statistical Manual for Mental Disorders (DSM), exposures to trauma encompass a broad array of contexts, from public health crises to armed conflict and displacement, terrorism, natural disasters, and chronic interpersonal and community violence. Reflecting this breadth of risk, the World Health Organization (WHO) World Mental Health Surveys report that more than 70% of adults have experienced at least one traumatic event, with approximately one third reporting four or more (Benjet et al., 2016). Thus, even if resilience is not ubiquitous when defined stringently across multiple domains and time points, the sheer frequency of exposure underscores the importance of identifying *why* and *how* some individuals maintain mental health despite exposure to traumatic experiences. Taken together, these considerations motivate a shift from questions of prevalence and measurement toward examining underlying mechanisms. Here, we focus on neurobiology because the core processes that govern adaptation to adversity, including threat appraisal, emotion regulation, and the stress-response systems, are instantiated in identifiable neural and endocrine circuits (Feder et al., 2019; McEwen et al., 2015; Russo et al., 2012; Yehuda et al., 2006). Neuroimaging allows these circuits to be quantified at both functional and structural levels.

## Neuroimaging perspectives on adversity and resilience

In keeping with this objective, neuroimaging research has examined the brain’s response to trauma and stress; yet the literature remains largely adversity-centric, with most work examining adversity-related correlates rather than resilience. A recent meta-analysis compiled 83 functional Magnetic Resonance Imaging (fMRI) studies involving individuals exposed to diverse adversities and found that adversity exposure was associated with heightened amygdala reactivity and reduced prefrontal cortex activity across emotional and cognitive tasks (Hosseini-Kamkar et al., 2023). These results are consistent with a large trauma-focused literature implicating fronto-limbic dysregulation as a neural signature of stress-related psychopathology (Gee et al., 2013; Koenigs and Grafman, 2009; Lanius et al., 2015; Nicholson et al., 2017; Rauch et al., 2000; Shin et al., 2006). In general, adversity-exposed groups tend to show neurobiological changes that may reflect vulnerability, including hyper-responsive threat circuitry coupled with compromised frontal regulatory control (Gee et al., 2013; McLaughlin et al., 2015; McLaughlin et al., 2019). While this adversity-centric approach has yielded important insights into risk mechanisms, it leaves open the complementary question of what distinguishes those who do not develop psychopathology despite exposure—that is, the neural correlates of resilience.

More recently, research has targeted resilience as the primary construct, evaluating whether resilient individuals show distinctive patterns of brain activity and connectivity. Several functional MRI studies report that trauma-exposed persons *without* PTSD, or high-risk individuals who remain healthy, recruit prefrontal and emotion-regulation networks more effectively than their non-resilient peers (Feder et al., 2009; Wu et al., 2013). Consistent with this, a recent systematic review and coordinate-based meta-analysis of 154 neuroimaging studies identified reliable associations of resilience with circuits centered on the bilateral amygdala and anterior cingulate cortex (Kuehn et al., 2025). Such convergences suggest that emotional-processing and regulatory systems may serve as general neural markers of resilient outcomes.

Nevertheless, functional findings outpace structural ones. In contrast to brain function, the structural correlates of resilience, including whether resilient individuals exhibit discernible differences in gray matter volume (GMV), remain comparatively under-studied and poorly understood. A few structural MRI investigations have reported structural correlates of resilience (see Supplementary Table 1), but results are mixed and often specific to particular risk contexts. Several reports link resilient outcomes to greater prefrontal and cingulate GMV (vmPFC, vlPFC/IFG, right middle frontal gyrus, ACC; Mangelsdorf, 2017; Pan et al., 2024; Kim et al., 2024; Burt et al., 2016; Amico et al., 2011; Filippi et al., 2021). Others implicate limbic, sensorimotor, posterior midline, and cerebellar regions, including the right amygdala, right postcentral gyrus, bilateral precuneus, calcarine cortex, and cerebellar vermis, with mixed directions and context-specific effects (Burhanoglu et al., 2021; Cornwell et al., 2023; You and Jackson, 2021; Chen et al., 2024; Kempton et al., 2009). Differences in risk context, analytic thresholds, and how resilience is operationalized, likely contribute to the inconsistency, emphasizing the need for a quantitative whole-brain meta-analysis. To date, no comprehensive meta-analysis has synthesized evidence for GMV differences associated with resilience, representing a notable gap in the neurobiology of resilience.

## Objectives

The objective of this study was to identify convergent GMV correlates of psychological resilience using a whole-brain coordinate-based meta-analysis of voxel-based morphometry (VBM) studies. Structural correlates were emphasized because they have been relatively neglected compared to functional ones. Unlike functional measures that may fluctuate with situational demands or transient states, structural markers such as GMV provide relatively stable indices of neural architecture, making them well-suited for identifying enduring correlates of resilience. To address inconsistencies across single studies and to detect potentially subtle effects, multilevel kernel density analysis (MKDA) was applied to synthesize reported GMV–resilience associations, as it has been shown to be robust in synthesizing neuroimaging findings across heterogeneous samples and methods (Wager et al., 2007, 2009). This approach aggregates evidence from all reported coordinates of GMV differences related to resilience without restricting analyses to *a priori* regions, enabling a whole-brain, data-driven test of spatial convergence. By delineating structural correlates, this meta-analysis is intended to complement functional models and refine neurobiological accounts of how some individuals maintain mental health in the face of hardship.

## Methods

### Search strategy

**Figure 1:**
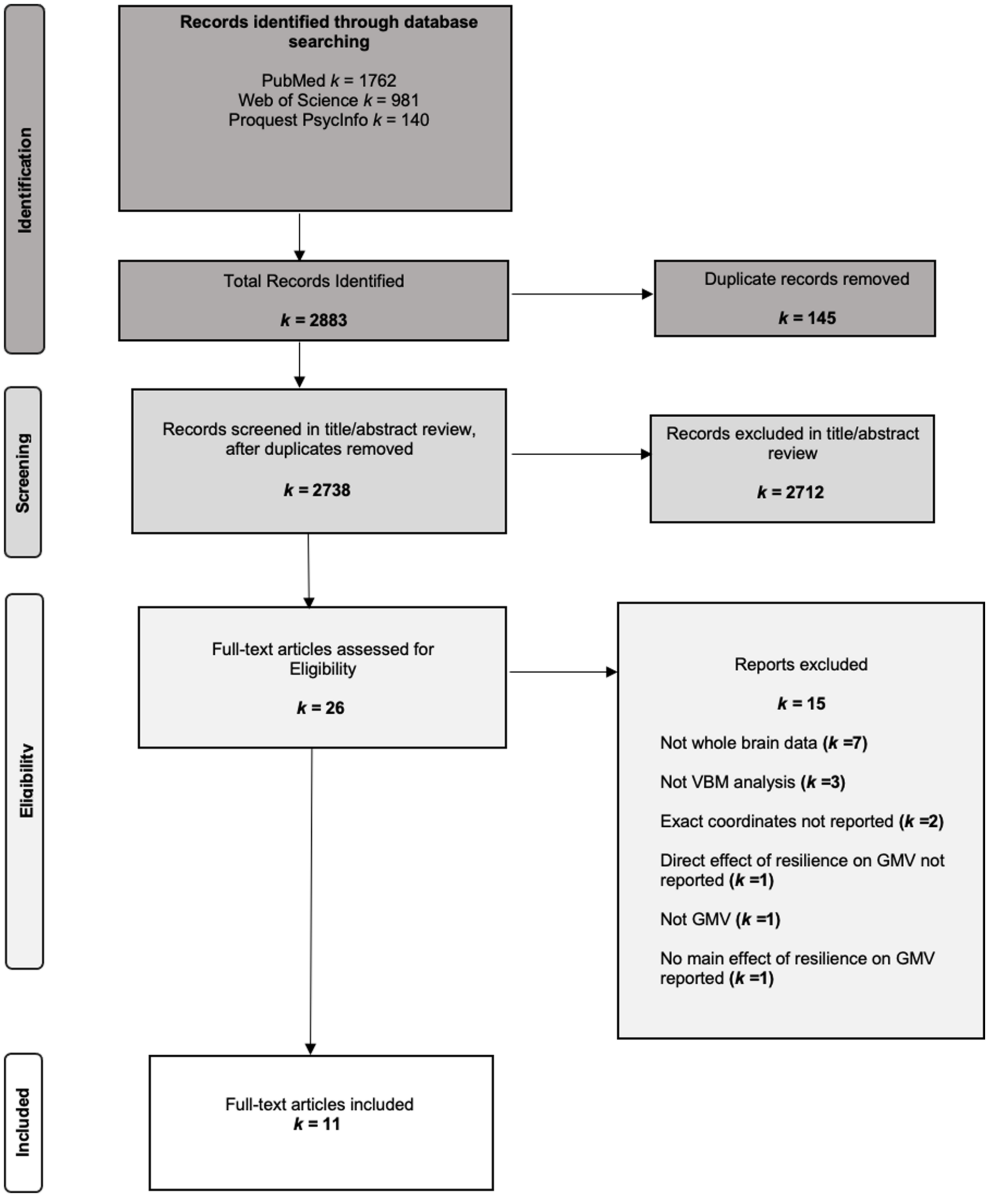
PRISMA flowchart of search yield.

We conducted systematic searches on January 11, 2025, in PubMed/MEDLINE, Web of Science, and ProQuest PsycINFO. We updated the database searches in June 2025 to ensure no eligible studies were missed. Searches combined resilience terms with neuroimaging terms using Boolean operators and database-specific controlled vocabulary (e.g., Medical Subject Headings (MeSH) terms where available). The resilience concept set was: Resilien* OR psychological resilien* OR *coping* OR *adaptation* OR *stress resistance*. For structural MRI, this set was intersected with: *Grey matter* OR *VBM* OR *voxel-based morphometry* OR *brain volume*. To ensure sensitivity, we also ran a parallel functional query—*Functional MRI* OR *task-based fMRI* OR *neural activation* OR *brain activation* OR *fMRI*—and retained records at screening if they potentially included structural outcomes; however, inclusion for the present study was restricted to VBM/GMV. Figure 1 details the full search and screening workflow.

### Eligibility criteria

Eligible studies met all of the following criteria: (a) human participants; (b) whole-brain voxel-based morphometry (VBM) analyses of gray matter volume (GMV); (c) an operationalization of psychological resilience (e.g., validated resilience scales or competence despite adversity) with a main effect relating resilience to GMV; and (d) reporting of stereotactic peak coordinates in Montreal Neurological Institute (MNI) or Talairach space. Reviews, meta-analyses, conference abstracts, case reports, animal studies, non-English publications, ROI-only analyses, studies without GMV outcomes, studies without a direct resilience–GMV effect, and reports that did not provide exact coordinates were excluded. Reasons for full-text exclusion and counts are summarized in Figure 1. The review adhered to the Preferred Reporting Items for Systematic Reviews and Meta-Analyses (PRISMA) guidelines and was pre-registered prior to analysis.^1^

### Study selection

Our search identified a total of 2,883 articles. After removal of 145 duplicates, 2,738 records underwent title/abstract screening; 2,712 were excluded. At full text review, 26 articles were assessed, and 11 met inclusion criteria. Among full-text exclusions (*k* = 15), reasons were: articles that did not report whole-brain data (*k* = 7), did not conduct a VBM analysis (*k* = 3), did not report exact coordinates (*k* = 2), did not report direct effect of resilience on GMV(*k* = 1), did not include GMV data (*k* = 1), or did not report main effect of resilience on GMV reported (*k* = 1). See Figure 1 (PRISMA). From the remaining 11 studies, we extracted sample characteristics, resilience operationalization, and all reported MNI/Talairach peak coordinates for resilience–GMV effects. If multiple models were reported, we prioritized whole-brain, corrected results.

### Statistical analysis

Peak stereotactic coordinates (x: left–right; y: posterior–anterior; z: inferior–superior) were extracted from the 11 included studies and coded by direction (greater GMV associated with higher resilience or resilient status; lower GMV associated with higher resilience). A meta-analysis was conducted in the Canlab MKDA toolbox, a coordinate-based framework that quantifies study-wise spatial convergence while treating each contrast as a random effect (Kober and Wager, 2010; Wager et al., 2007, 2009). In MKDA, all peaks from a given contrast are merged into a single contrast-indicator map so that each contrast can contribute at most one activation per voxel; this prevents studies that report many foci from exerting disproportionate influence (Kober and Wager, 2010; Wager et al., 2007, 2009). Contrasts are weighted by the square root of sample size and studies using fixed-effects analyses are down-weighted, ensuring that papers with multiple significant clusters do not receive extra weight beyond their single contrast-level contribution (Kober and Wager, 2010; Wager et al., 2007, 2009). Talairach coordinates were converted to MNI space using the Lancaster icbm2tal transformation when required.

For each contrast, reported peaks were modeled as spherical kernels and combined into study indicator maps; maps were then weighted by sample size and aggregated to yield proportion-of-studies density maps (Wager et al., 2007). We used a 25-mm kernel radius. This relatively broad kernel was chosen *a priori* to accommodate inter-study spatial uncertainty inherent to VBM, such as between-study differences in preprocessing and normalization and typical smoothing of ~8–12 mm Full Width at Half Maximum (FWHM). This approach also accounts for the heterogeneous operationalizations of resilience, and prioritizes sensitivity to regional convergence given the small number of available studies. In other words, the analysis trades fine spatial specificity for reduced type-II errors in an exploratory synthesis.

Statistical significance was evaluated with 10,000 Monte Carlo permutations that preserved the number of peaks per study while randomly relocating them within gray matter to generate a null distribution of the maximum statistic. Voxels or clusters were considered significant if the observed weighted proportion of contributing contrasts exceeded the 95th percentile of the corresponding null distribution, yielding family-wise error–controlled thresholds (*p* < 0.05 FWE). In practical terms, significance indicates spatial convergence of resilience-related GMV effects across studies that is unlikely to arise from random placement of foci (Wager et al., 2007, 2009). Thus, the resulting meta-analytic map represents regions where GMV is consistently related to resilience across studies. All analyses were implemented in MATLAB R2025a using the Canlab MKDA toolbox.^2^

## Results

**Figure 2:**
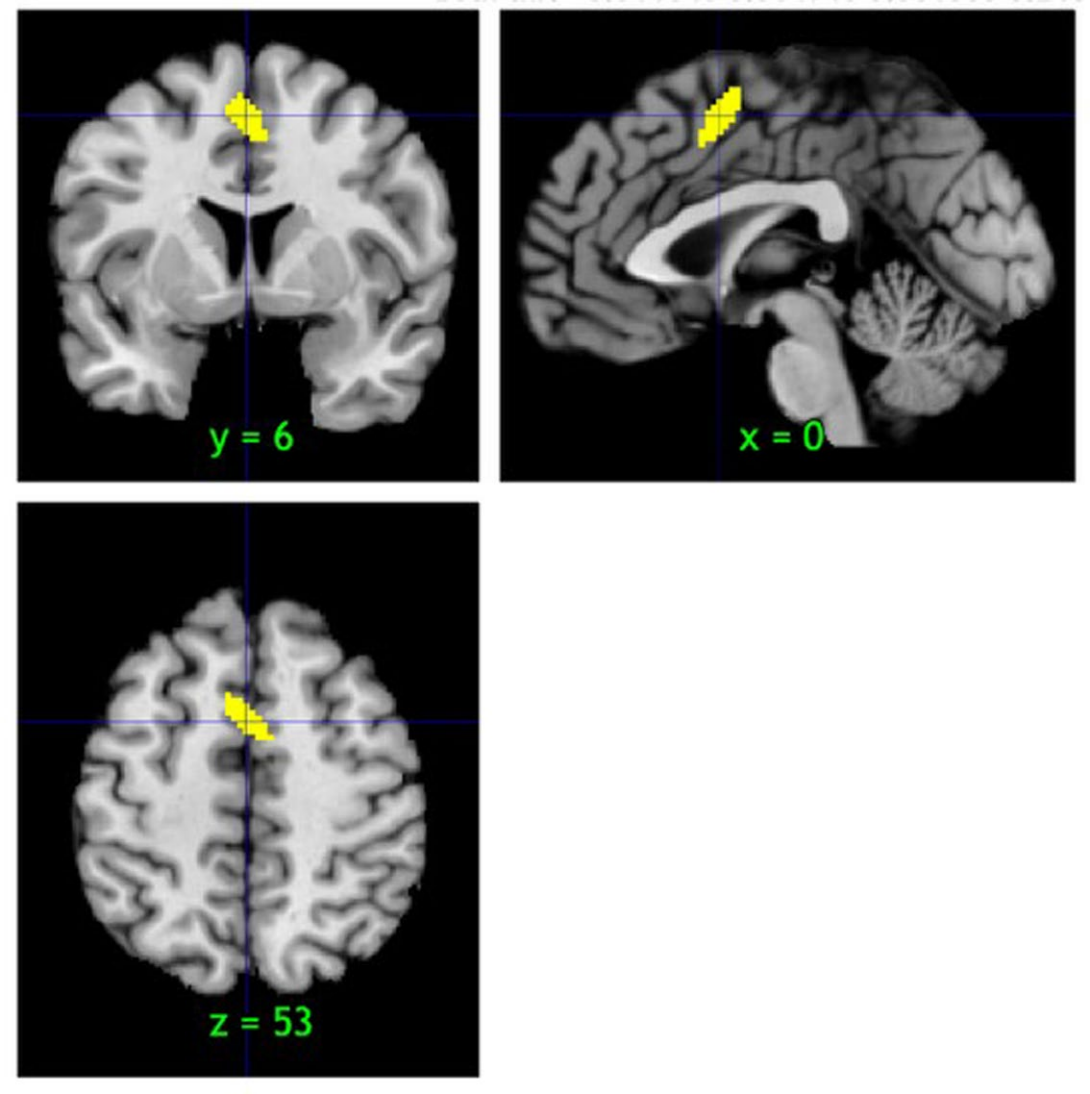
Convergence of peaks across studies showing greater GMV correlates of resilience. MKDA random-effects analysis of 11 whole-brain VBM studies (47 coordinates) identified a single cluster of cross-study convergence extending from the superior frontal gyrus through the medial frontal gyrus into the cingulate gyrus (familywise error rate–corrected *p* < 0.05). The peak of this cluster was located at MNI (*x* = 0, *y* = 6, *z* = 53).

Across the 11 voxel-based morphometry studies, the pooled sample comprised 2,993 participants and 47 peak coordinates associated with GMV in relation to resilient outcomes (Supplementary Table 1). To test for spatial convergence, we conducted an MKDA on the coordinates that reported associations between greater GMV and resilience only. This analysis identified one reliable cluster on the medial frontal wall, extending from the superior frontal gyrus (MNI x = 0, y = 6, z = 53) and medial frontal gyrus (x = 5, y = 3, z = 52) to the cingulate gyrus (x = 5, y = 7, z = 42), significant at familywise-error–corrected *p* < 0.05 (labels via WFU PickAtlas; Figure 2). By contrast, a complementary meta-analysis of reduced GMV correlates could not be performed because only four peaks were available across studies, precluding a stable test of convergence. At the single-study level, additional foci were distributed across prefrontal, limbic, sensorimotor, posterior midline, and cerebellar regions; however, only the medial frontal–cingulate region demonstrated cross-study convergence at corrected thresholds, as shown in Figure 2. We did not exclude small-sample or lenient-threshold studies because with only *k* = 11 studies removing even a few contrasts would materially reduce power and destabilize the permutation-based FWE thresholds in MKDA (Wager et al., 2007, 2009). As a check on robustness, a 15-mm kernel sensitivity analysis reproduced the same locus with reduced extent (peak x = -1, y = 7, z = 52; see Supplementary material).

## Discussion

This coordinate-based meta-analysis provides the first quantitative synthesis of VBM studies examining the structural correlates of psychological resilience. Across 11 whole-brain studies (*n* = 2,993) and 47 peak coordinates, we identified a single familywise-error–corrected cluster of convergence along the medial frontal wall spanning the superior/medial frontal gyrus into the dorsal cingulate gyrus. Greater GMV in this region was associated with more resilient outcomes, whereas the available data were insufficient to analyze reduced-GMV effects across studies. These findings delineate a putative structural substrate for resilience that complements behavioral and functional evidence. Given the prevalence of traumatic experiences, advancing the scientific understanding of resilience is of critical importance. Taken together, these results delineate key neurological structures that may represent core nodes associated with resilience.

### The medial frontal–cingulate circuit as a resilience node

The medial frontal gyrus and dorsal anterior cingulate cortex (dACC) are widely regarded as core nodes for higher-order control (Bush et al., 2002). Classic conflict-monitoring accounts position the dACC as a detector of response conflict that signals the need for increased top-down control (Botvinick et al., 2001; Carter et al., 1998). More recent computational formulations specify that the dACC estimates the expected value of control to guide how control is allocated (Shenhav et al., 2013). Consistent with these accounts, human neuroimaging reviews show reliable engagement of medial frontal and dorsal cingulate regions during performance monitoring and adaptive control across a range of tasks (Ridderinkhof et al., 2004; Dosenbach et al., 2008). Importantly, the same medial frontal territory contributes to affective control with the dACC and dorsomedial prefrontal cortex being engaged during reappraisal of aversive stimuli, resolution of emotional conflict, or down-regulation of negative affect (Etkin et al., 2011; Ochsner et al., 2012; Shackman et al., 2011; see also Buhle et al., 2014).

The medial frontal–cingulate region is functionally heterogeneous, with subregions supporting different facets of control. Dorsal areas including the superior frontal gyrus and dorsal anterior/mid-cingulate are reliably engaged by cognitive control demands such as conflict monitoring, performance adjustment, and sustained task control (Botvinick et al., 2004; Dosenbach et al., 2008; Shackman et al., 2011). By contrast, ventral/rostral anterior cingulate and medial prefrontal regions are preferentially implicated in affective control and emotion regulation, including top-down modulation of limbic responses (Bush et al., 2000; Ochsner and Gross, 2005; Etkin et al., 2011). This division of labor (dorsal for cognitive control, ventral for affective control) provides a mechanistic account of how resilience may be supported by coordinated top-down regulation across these midline systems under stress.

Indeed, converging evidence from animal models further indicates that the medial prefrontal cortex exerts inhibitory control over stress-responsive limbic circuits; animals that can control aversive stimulation show prefrontal engagement and are protected against learned helplessness, whereas yoked animals without control exhibit persistent stress responses (Maier et al., 2006). Complementing these observations, multimodal human imaging links higher dispositional resilience to stronger structural and functional coupling within frontal control networks that include medial frontal and cingulate hubs (Hsieh et al., 2021). Thus, our meta-analytic convergence of greater gray-matter volume increases along the medial frontal gyrus into the dorsal cingulate is consistent with the view that a more robust frontal-midline architecture supports adaptive appraisal, reappraisal, and regulation in the face of adversity.

### Relation to adversity-centric neuroimaging studies

A previous functional meta-analysis of adversity (Hosseini-Kamkar et al., 2023) showed an association between adversity exposure and both heightened amygdala reactivity and reduced prefrontal engagement across tasks, which is consistent with risk for psychopathology. The current structural synthesis, centered on resilience, points in the opposite direction with increased GMV in medial prefrontal and anterior cingulate territory. A parsimonious interpretation is that resilient outcomes are supported by anatomy that sustains effective regulatory control over limbic systems that are otherwise prone to hyperreactivity following adversity exposure (Gee et al., 2013; Koenigs and Grafman, 2009; Rauch et al., 2000; Shin et al., 2006). Taken together, this pattern suggests a division between risk markers, namely limbic hyperreactivity with attenuated prefrontal recruitment, and protection markers, reflected in greater medial frontal–cingulate volume. However, this risk versus protection distinction does not yield uniform single-study results, and considerable heterogeneity remains across VBM reports. Effects reported in limbic, sensorimotor, posterior midline, and cerebellar regions likely reflect context-specific demands, measurement differences, or compensatory processes that vary by sample and adversity type, and with limited sample sizes, they do not reproduce reliably across studies. When evidence is pooled under whole-brain familywise-error control, the only locus that consistently exceeds meta-analytic thresholds is the medial frontal–cingulate hub, which points to a common structural denominator that generalizes across studies and aligns with models of fronto-limbic regulatory control (Shackman et al., 2011).

### Advantages of MKDA for a heterogeneous literature

From a methodological standpoint, MKDA is designed to determine convergent findings across a heterogeneous literature. The analysis treats each independent contrast as the primary observation rather than each reported coordinate, which prevents studies that list many peaks from carrying disproportionate weight (Wager et al., 2007). Moreover, familywise-error rate control is obtained by Monte Carlo simulation of the null distribution, yielding conservative thresholds that are appropriate for exploratory syntheses with few studies (Wager et al., 2007, 2009). A further advantage concerns interpretability and generalization. Because the MKDA statistic indexes the proportion of studies that show an effect near a location, a significant cluster implies that a non-zero fraction of future studies from the same domain should also report an effect in that region (Wager et al., 2007, 2009). By comparison, Activation Likelihood Estimation (ALE) aggregates the spatial coincidence of peaks to form a density map. Although modern ALE variants reduce within-study clustering, the ALE statistic does not directly quantify study-wise prevalence, and clusters can still be influenced by a few studies that contribute many foci. For questions that emphasize robustness across independent samples, MKDA therefore offers a more direct statement about reproducibility (Wager et al., 2007, 2009).

### Limitations and future directions

Although these results offer preliminary evidence for convergent GMV correlates of resilience in the medial frontal gyrus and anterior cingulate cortex, they should be interpreted cautiously given several limitations. First, the analysis remains exploratory, as only 11 studies met inclusion criteria and we adopted a relatively large 25 mm kernel radius, sacrificing spatial precision to accommodate for variability in VBM preprocessing and heterogeneous resilience definitions across studies. Second, the limited number of studies reporting reduced-GMV effects precluded a balanced examination of directionality. In addition, because coordinate-based meta-analyses depend on selectively reported peak foci, and because only four peaks were available for reduced-GMV effects, the current evidence base is vulnerable to small-study and positive-finding reporting bias that may over-represent increases in GMV relative to decreases. We therefore refrain from drawing directional conclusions about GMV reductions and emphasize the need for preregistered analyses and sharing of unthresholded statistical maps to enable image-based meta-analyses that more directly assess reporting bias. Moreover, formal tests for publication bias are not available for MKDA; accordingly, we did not perform a statistical test for publication bias. Because MKDA depends on reported peak foci, we note the possibility that reporting practices could influence convergence and reiterate the need for preregistration and sharing of unthresholded statistical maps to support future image-based meta-analyses (Wager et al., 2007, 2009). Third, resilience definitions and measurement varied widely across studies, ranging from proxy definitions (e.g., health in high-risk relatives) to trait questionnaires, and rarely assessed multiple outcome domains; such heterogeneity likely increases between-study variance and may obscure additional convergent regions. Moreover, the data were cross-sectional, limiting causal inference about whether larger medial prefrontal GMV precedes adversity, reflects experience-dependent plasticity, or represents pre-existing individual differences that confer resilience. In other words, it remains unclear whether structural differences in medial prefrontal GMV serve as antecedent protective factors, emerge as a consequence of exposure, or reflect a dynamic interplay between both processes. Finally, our meta-analysis included datasets from combined community and clinical/high-risk samples; however, with only 11 whole-brain VBM studies, MKDA subgroup tests by clinical status were not feasible, therefore, we cannot determine whether clinical heterogeneity contributed to the medial frontal–cingulate convergence.

Future work should close these gaps by using longitudinal designs that measure brain structure before and after adverse experiences to capture temporal dynamics and plasticity. Studies should also adopt multidimensional definitions of resilience that integrate indicators of mental health, well-being, and psychosocial functioning. Methodologically, harmonizing VBM pipelines and sharing raw data would enable more precise image-based meta-analyses and pooled mega-analyses. Analyses should be stratified by adversity type (e.g., threat vs. deprivation) and by severity to test whether and how different experiences interact with neurobiology to predict resilience. Finally, multimodal imaging studies should examine whether larger medial frontal and anterior cingulate cortex volumes prospectively predict more effective top-down regulation during stress, and whether targeted interventions (e.g., cognitive reappraisal training, neuromodulation) can strengthen structural or functional markers of resilience (Charney, 2004; Feder et al., 2019; Russo et al., 2012).

### Clinical and translational implications

Identification of a structural correlate in the medial frontal and dorsal cingulate has practical implications. This region is engaged by cognitive reappraisal and mindfulness and is accessible to noninvasive brain stimulation (Hölzel et al., 2011; Opialla et al., 2015; Marques et al., 2019). In the near term, gray matter volume in this territory could help stratify participants and track change in intervention studies, with pre to post designs testing whether targeted training or neuromodulation strengthens this architecture (Feder et al., 2019). These applications remain provisional until longitudinal and mechanistic work links structural change in this region to improved regulation and clinical outcomes.

## Conclusion

In a literature characterized by contested definitions, heterogeneous single-study reports, and a predominant focus on functional rather than structural markers, the present MKDA offers the first quantitative synthesis of gray matter correlates of psychological resilience. We demonstrate that greater GMV in a medial frontal–dorsal cingulate cluster emerges as the sole convergent structural signature across existing studies. This finding supports theories positing that a robust fronto-cingulate substrate underlies adaptive control and regulation in the face of adversity. Thus, by highlighting a consistent anatomical correlate, the present meta-analysis lays the initial groundwork for targeted research into the neurobiology of resilience. These findings also motivate longitudinal and multimodal analyses of whether medial frontal–cingulate structure prospectively supports adaptive responding and if it can be strengthened through behavioral or neuromodulatory interventions in populations exposed to trauma.

## Data Availability

The original contributions presented in the study are included in the article/Supplementary material, further inquiries can be directed to the corresponding author.

## Footnotes

1
https://osf.io/7nb6k/

2
https://github.com/canlab/Canlab_MKDA_MetaAnalysis
