# Supplementary material for: Gray matter volume correlates of resilience: a coordinate-based meta-analysis
Source: Front Psychol. 2026 Mar 24;17:1691912. doi: 10.3389/fpsyg.2026.1691912 (PMC13053268; doi:10.3389/fpsyg.2026.1691912)
Supplement: Supplementary file 1 [file Data_Sheet_1.docx]

**Supplementary material. Publications that have provided evidence for the Utility Model (ordered by year and alphabetically)**

**1. Levels of use of the General Utility Conceptual Model**

de la Fuente J, González-Torres MC, Aznárez-Sanado M, Martínez-Vicente JM, Peralta-Sánchez FJ and Vera MM (2019). Implications of Unconnected Micro, Molecular, and Molar Level Research in Psychology: The Case of Executive Functions, Self-Regulation, and External Regulation. *Front. Psychol. 10*,1919. doi: 10.3389/fpsyg.2019.01919

de la Fuente J and Martínez-Vicente JM (2024). Conceptual Utility Model for the Management of Stress and Psychological Wellbeing, CMMSPW™ in a university environment: theoretical basis, structure and functionality. *Front. Psychol. 14*:1299224. doi: 10.3389/fpsyg.2023.1299224

de la Fuente J and Martínez-Vicente JM (2024). *Modelo Conceptual para la para Gestión del Estrés y del Bienestar Psicológico, MCGEBP ®: Fundamentos, Estructura y Funcionalidad.* Ed: Independently published (Amazon: Serie Education & Psychology I+D+I).

de la Fuente J and Martínez-Vicente JM (2024). *Conceptual Model for the Management of Stress and Psychological Wellbeing CMMSPW®: Foundations, Structure and Functionality.* Ed: Independently published (Amazon: Serie Education & Psychology I+D+I).

de la Fuente-Arias, J., Martínez-Vicente, J. M., Peralta Sánchez, F. J., Pachón-Basallo, M., & Garzón-Umerenkova, A. (2025). Experience Academic Wellbeing Index as Predictor of Psychological Wellbeing and Flourishing. *New Directions for Child and Adolescent Development, 2025*(1), 8862531. <https://doi.org/10.1155/cad/8862531>

**2. e-Stress Coping Tool**

de la Fuente, J., López, M., Zapata, L., Sollinas, G., & Fadda, S. (2015). Improving mental Health trough and online self-assessment and self-help e-Utility in university Students. *Progress in education, 33*, 63-74. NY: Nova Publiher Incorp.

de la Fuente-Arias, J., Martínez-Vicente, JM, Peralta-Sánchez, FJ. y Pachón-Basallo, M. (2024). M. *La Utilidad e-Afrontamiento® como herramienta tecnológica para aplicar el Modelo de Utilidad Conceptual para la Gestión del Estrés y el Bienestar Psicológico, MCGEBP®.* Memorias de la Vigésima Tercera Conferencia Iberoamericana en Sistemas, Cibernética e Informática (CISCI 2024) pp. 279-283. https://doi.org/10.54808/CISCI2024.01.279

**3. Variables inherent to the model**

**3.1. Presage variables:**

**3.1.1. Individual factors**

**Gender and Years**

de la Fuente, J., Cardelle-Elawar, M., Martínez-Vicente, J. M., Zapata, L., & Peralta, F. J. (2013). Gender as a determining factor in the coping strategies and resilience of university students. *Handbook of Academic Performance*, 205-217. NY: Nova Publishers Incorporation.

López-Madrigal, C., de la Fuente, J., García-Manglano, J., Martínez-Vicente, J. M., Peralta-Sánchez, F. J., & Amate-Romera, J. (2021). The role of gender and age in the emotional well-being outcomes of young adults*. International journal of environmental research and public health, 18*(2), 522. <https://doi.org/10.3390/ijerph18020522>

de la Fuente, J., Díaz de Terán-Velasco, M. C., Ladrón de Guevara-Pascual, B., Aguiló-Pastrana, A., Mora de la Rosa, C., Camaño, R., ... & Rodríguez-Lago, B. (2025). Gender difference as protective and risk factors in the Competence for Living in Equality (CLE) in adolescents. *Cogent Education, 12*(1), 2577903. <https://doi.org/10.1080/2331186X.2025.2577903>

**Personality (Big Five)**

de la Fuente, J., Paoloni, P., Kauffman, D., Yilmaz Soylu, M., Sander, P., & Zapata, L. (2020). Big five, self-regulation, and coping strategies as predictors of achievement emotions in undergraduate students. *International journal of environmental research and public health, 17*(10), 3602**.** <https://doi.org/10.3390/ijerph17103602>

de la Fuente, J., Malpica-Chavarria, E. A., Garzón-Umerenkova, A., & Pachón-Basallo, M. (2021). Effect of personal and contextual factors of regulation on academic achievement during adolescence: the role of gender and age*. International Journal of Environmental Research and Public Health, 18*(17), 8944.https://doi.org/10.3390/ijerph18178944

de la Fuente, J., Sander, P., Garzón-Umerenkova, A., Urien, B., Pachón-Basallo, M., & Luis, E. O. (2024). The big five factors as differential predictors of self-regulation, achievement emotions, coping and health behavior in undergraduate students. *BMC Psychology, 12*, 267. doi: 10.1186/s40359-024-01768-9

de la Fuente, J. y Zapata, L. (2026). *Personal Self-regulation, learning Appraches and Coping Strategies, In University teaching and Learning Processess with Stress*. Ed: Independently published (Amazon: Serie Education & Psychology I+D+I).

**Positive vs Negative Affects**

de la Fuente-Arias, J., Franco-Justo, C. F., & Mañas, I. M. (2010). Efectos de un programa de entrenamiento en conciencia plena (mindfulness) en el estado emocional de estudiantes universitarios. *Estudios sobre educación, 19*, 31-52.

**Personal Self-regulation Behavior**

de la Fuente-Arias, J., Sánchez, F. J. P., & Roda, M. D. S. (2009). Autorregulación personal y percepción de los comportamientos escolares desadaptativos. *Psicothema, 21*(4), 548-554.

de la Fuente, J., & Cardelle-Elawar, M. (2011). Personal self-regulation and coping style in university students. LB, and RA Nichelson (Eds.), Psychology of individual Differences, 171-182. NY: Nova Incorporation Publishers.

de la Fuente, J., Zapata, L., Martínez-Vicente, J. M., Sander, P., & Putwain, D. (2014). Personal self-regulation, self-regulated learning and coping strategies, in university context with stress. In Metacognition: *Fundaments, applications, and trends: A profile of the current state-of-the-art* (pp. 223-255). Cham: Springer International Publishing.

Pichardo, C., Justicia, F., de la Fuente, J., Martínez-Vicente, J. M., & García-Berbén, A. B. (2014). Factor structure of the self-regulation questionnaire (SRQ) at Spanish Universities*. The Spanish Journal of Psychology, 17*, E62. doi:10.1017/sjp.2014.63

Zapata, L., de la Fuente, J., Martínez-Vicente, J. M., González-Torres, M. C., & Artuch, R. (2014). Relations between the personal self-regulation and learning approach, coping strategies, and self-regulation learning, in university students (process*). International Journal of Developmental and Educational Psychology, 4*(1), 175-186.

Zapata, L., de la Fuente, J. Sander, P. & Putwin, D. (2024). Personal self-regulation as a variable student (presage). (2014). *International Journal of Developmental and Educational Psychology, 4*(1), 165-174. https://doi.org/10.17060/ijodaep.2014.n1.v4.600

de la Fuente J, Zapata L, Martínez-Vicente JM, Sander P and Cardelle-Elawar M (2015). The role of personal self-regulation and regulatory teaching to predict motivational-affective variables, achievement, and satisfaction: a structural model. *Front. Psychol. 6:*399. doi: 10.3389/fpsyg.2015.00399

de la Fuente, J., López-García, M., Mariano-Vera, M., Martínez-Vicente, J. M., & Zapata, L. (2017). Personal self-regulation, learning approaches, resilience and test anxiety in psychology students. *Estudios sobre educación, 32*, 9-26.

de la Fuente J, Sander P, Martínez-Vicente JM, Vera M, Garzón A and Fadda S (2017). Combined Effect of Levels in Personal Self-Regulation and Regulatory Teaching on Meta-Cognitive, on Meta-Motivational, and on Academic Achievement Variables in Undergraduate Students. *Front. Psychol. 8:*232. doi: 10.3389/fpsyg.2017.00232

Garzón-Umerenkova A, de la Fuente-Arias J, Martínez-Vicente JM, Zapata-Sevillano L, Pichardo MC and García-Berbén AB (2017). Validation of the Spanish Short Self-Regulation Questionnaire (SSSRQ) through Rasch Analysis. *Front. Psychol. 8:*276. doi: 10.3389/fpsyg.2017.00276

Garzón-Umerenkova A, de la Fuente J, Amate J, Paoloni PV, Fadda S and Pérez JF (2018). A Linear Empirical Model of Self-Regulation on Flourishing, Health, Procrastination, and Achievement, Among University Students. *Front. Psychol. 9,*536. doi: 10.3389/fpsyg.2018.00536

Pichardo MC, Cano F, Garzón-Umerenkova A, de la Fuente J, Peralta-Sánchez FJ and Amate-Romera J (2018). Self-Regulation Questionnaire (SRQ) in Spanish Adolescents: Factor Structure and Rasch Analysis. *Front. Psychol. 9*:1370. doi: 10.3389/fpsyg.2018.01370

**Internal- vs Externally- Behavioral Regulation**

de la Fuente J, Pachón-Basallo M, Martínez-Vicente JM, Peralta-Sánchez FJ, Garzón-Umerenkova A and Sander P (2022). Self- vs. External-Regulation Behavior Scale ^TM^ in different psychological contexts: A validation study. *Front. Psychol. 13:*922633. doi: 10.3389/fpsyg.2022.922633

de la Fuente J, Kauffman DF and Boruchovitch E (2023). Editorial: Past, present and future contributions from the social cognitive theory (Albert Bandura). *Front. Psychol. 14:*1258249. doi: 10.3389/fpsyg.2023.1258249

de la Fuente, J. (2023). *Escalas para la Evaluación de la Regulación Comportamental Interna vs Externa, en diferentes Contextos Psicológicos: Versión multilingüe.* Ed: Independently published (Amazon: Serie Education & Psychology I+D+I).

de la Fuente. J. (2023). *Scales for the Assessment of Internal-External Regulation in different Psychological Contexts: Multilingual version.* Ed: Independently published (Amazon: Serie Education & Psychology I+D+I).

de la Fuente, J. (2024a). *Escalas para la Evaluación de la Regulación Comportamental Interna vs Externa, en diferentes Contextos Psicológicos: Versión multilingüe.* Ed: Independently published (Amazon: Serie Education & Psychology I+D+I).

de la Fuente, J. (2024b). *Scales for the Assessment of Internal-External Regulation in different Psychological Contexts: Multilingual version*. Ed: Independently published (Amazon: Serie Education & Psychology I+D+I).

de la Fuente, J. & Kauffman, D.F. (Eds) (2025*). Theory of Self- vs Externally-Regulated Behavior. Applicability to Educational, Clinical, Health, and Organizational Psychology Contexts.* NY: Nova Science Publishers. <https://doi.org/10.52305/LUGJ1847>

de la Fuente, J. (2025). *El Índice Combinado de Regulación Comportamental Interno-Externo (ICRIE).* Ed: Independently published (Amazon: Serie Education & Psychology I+D+I).

**Psycho-Educational context**

de la Fuente-Arias J (2017). Theory of Self- vs. Externally-Regulated LearningTM: Fundamentals, Evidence, and Applicability. *Front. Psychol. 8:*1675. doi: 10.3389/fpsyg.2017.01675

de la Fuente J, Sander P, Kauffman DF and Yilmaz Soylu M (2020). Differential Effects of Self- vs. External-Regulation on Learning Approaches, Academic Achievement, and Satisfaction in Undergraduate Students. *Front. Psychol. 11:*543884. doi: 10.3389/fpsyg.2020.543884

de la Fuente, J., Malpica-Chavarria, E. A., Garzón-Umerenkova, A., & Pachón-Basallo, M. (2021). Effect of personal and contextual factors of regulation on academic achievement during adolescence: the role of gender and age. *International Journal of Environmental Research and Public Health, 18*(17), 8944.https://doi.org/10.3390/ijerph18178944

de la Fuente J, Martínez-Vicente JM, Santos FH, Sander P, Fadda S, Karagiannopoulou E, Boruchovitch E and Kauffman DF (2022). Advances on Self-Regulation Models: A New Research Agenda Through the SR vs ER Behavior Theory in Different Psychology Contexts. *Front. Psychol. 13*:861493. doi: 10.3389/fpsyg.2022.861493

de la Fuente J, Kauffman DF, Dempsy MS and Kauffman Y (2022) Editorial: Coronavirus disease (COVID-19): Psychoeducational variables involved in the health emergency. *Front. Psychol. 13*:961261. doi: 10.3389/fpsyg.2022.961261

López-Madrigal, C., García-Manglano, J., & de la Fuente Arias, J. (2022). A path analysis model of self-vs. educational-context-regulation as combined predictors of well-being in Spanish college students. *International Journal of Environmental Research and Public Health, 19*(16), 10223.

Pachón-Basallo M, de la Fuente J, González-Torres MC, Martínez-Vicente JM, Peralta-Sánchez FJ and Vera-Martínez MM (2022). Effects of factors of self-regulation vs. factors of external regulation of learning in self-regulated study. *Front. Psychol. 13*:968733. doi: 10.3389/fpsyg.2022.968733

de la Fuente, J. y Martínez-Vicente, JM (2024). *El Índice Experiencia de Bienestar Escolar o Académico (IEBEA) como indicador de la Calidad Educativa.* Ed: Independently published (Amazon: Serie Education & Psychology I+D+I).

de la Fuente, J. (2025a). *El Índice Combinado de Regulación Comportamental Interna y Externa(ICRIE): Estructura, evidencia y aplicabilidad.* Ed: Independently published (Amazon: Serie Education & Psychology I+D+I).

de la Fuente, J. (2025b). *El Índice Combinado de Regulación Comportamental en el Proceso de Enseñanza-Aprendizaje, ICREA®.*  Ed: Independently published (Amazon: Serie Education & Psychology I+D+I).

de la Fuente, J. (2025b). *El Índice Combinado de Salud Académica (Física y Psicológica), ICSA®.* Ed: Independently published (Amazon: Serie Education & Psychology I+D+I).

**Psycho-Health context**

Pachón-Basallo, M., de la Fuente, J., & Gonzáles-Torres, M. C. (2021). Regulation/non-regulation/dys-regulation of health behavior, psychological reactance, and health of university undergraduate students*. International Journal of Environmental Research and Public Health, 18*(7), 3793. doi: 10.3389/fpsyt.2021.600240

**Psycho-Organizational psychology**

Urien, B., Pignatta, S., Fadda, S., & de la Fuente, J. (2025). REGULACION PERSONAL Y CONTEXTUAL (SRO-ERO), PCTA, ENGAGEMENT-BOURNOUT AND PSYCHOLOGICAL WELL-BEING: IMPLICACIONES ORGANIZACIONALES [PERSONAL AND CONTEXTUAL REGULATION ORGANIZATIONAL (SRO-ERO), ENGAGEMENT- BOURNOUT, AND PSYCHOLOGICAL WELL-BEING: ORGANIZATIONAL IMPLICATIONS]. In review.

**1.2. Contextual factor**

**1.2.1. Internal- vs Externally- Behavioral Regulation**

**Psycho-Educational context**

de la Fuente-Arias J (2017) Theory of Self- vs. Externally-Regulated LearningTM: Fundamentals, Evidence, and Applicability. *Front. Psychol. 8:*1675. doi: 10.3389/fpsyg.2017.01675

de la Fuente J, Sander P, Kauffman DF and Yilmaz Soylu M (2020). Differential Effects of Self- vs. External-Regulation on Learning Approaches, Academic Achievement, and Satisfaction in Undergraduate Students. *Front. Psychol. 11:*543884. doi: 10.3389/fpsyg.2020.543884

de la Fuente, J., Malpica-Chavarria, E. A., Garzón-Umerenkova, A., & Pachón-Basallo, M. (2021). Effect of personal and contextual factors of regulation on academic achievement during adolescence: the role of gender and age. *International Journal of Environmental Research and Public Health, 18*(17), 8944.https://doi.org/10.3390/ijerph18178944

de la Fuente J, Martínez-Vicente JM, Santos FH, Sander P, Fadda S, Karagiannopoulou E, Boruchovitch E and Kauffman DF (2022). Advances on Self-Regulation Models: A New Research Agenda Through the SR vs ER Behavior Theory in Different Psychology Contexts. *Front. Psychol. 13*:861493. doi: 10.3389/fpsyg.2022.861493

de la Fuente J, Lecuona-López L, Pachón-Basallo M, San Martín-Íñiguez L and Blanco-Sarto P (2024) Addressing the links between and internal vs. external regulation factors, achievement emotions and gender in problematic use of ICT at university. *Front. Psychol. 15*:1382016. doi: 10.3389/fpsyg.2024.1382016

**Psycho-Health context**

de la Fuente, J., Zapata, L., & Martínez-Vicente, J. M. (2016). Effects of level of personal self-regulation and different contexts of stress on coping strategies in higher education. *Academic Performance: Student Expectations, Environmental Factors and Impacts on Health,* 77-90. NY: Nova Publishers Incorp.

Pachón-Basallo, M., de la Fuente, J., & Gonzáles-Torres, M. C. (2021). Regulation/non-regulation/dys-regulation of health behavior, psychological reactance, and health of university undergraduate students*. International Journal of Environmental Research and Public Health, 18*(7), 3793. doi: 10.3389/fpsyt.2021.600240

**Psycho-Organizational psychology**

Urien, B., Pignatta, S., Fadda, S., & de la Fuente, J. (2025). REGULACION PERSONAL Y CONTEXTUAL (SRO-ERO), PCTA, ENGAGEMENT-BOURNOUT AND PSYCHOLOGICAL WELL-BEING: IMPLICACIONES ORGANIZACIONALES [PERSONAL AND CONTEXTUAL REGULATION ORGANIZATIONAL (SRO-ERO), ENGAGEMENT- BOURNOUT, AND PSYCHOLOGICAL WELL-BEING: ORGANIZATIONAL IMPLICATIONS]. In review.

**1.2.2. Family Support**

Balaguer, A., Benítez, E., de la Fuente, J., & Osorio, A. (2021). Maternal and paternal parenting styles as a whole: validation of the simple form of the Parenting Style Evaluation Scale. *Anales de Psicología/Annals of Psychology, 37*(1), 77-87. <https://doi.org/10.6018/analesps.408171>

Balaguer, Á., Benítez, E., de la Fuente, J., & Osorio, A. (2022). Structural empirical model of personal positive youth development, parenting, and school climate*. Psychology in the Schools, 59*(3), 451-470. DOI: 10.1002/pits.22620

**2. Variables of process**

**2.1. Individual: Competence for management of stress and weel-being academic**

de la Fuente, J. (2025). *Competencia para estudiar, aprender y rendir bajo estrés: Guía para estudiantes y opositores.* Segunda Edición. Ed: Independently published (Amazon: Serie Education & Psychology I+D+I).

de la Fuente, J. (2025). COMPETENCY FOR STUDYING, LEARNING AND PERFORMING UNDER STRESS: SELF-HELP GUIDE FOR UNIVERSITY STUDENTS, GRADUATES AND PROFESSIONAL EXAMINATION CANDIDATES. Ed: Independently published (Amazon: Serie Education & Psychology I+D+I).

**2.1.1. Conceptual Sub-competence**

**· Learning Approaches**

de la Fuente-Arias, J.; Pichardo, M.C.; Justicia, F.; García-Berbén, A. B. (2008). Enfoques de aprendizaje, autorregulación y rendimiento en tres universidades europeas. *Psicothema, 20* (4), 705-711.

Justicia, F., Pichardo, M. C., Cano, F., García-Berbén, A. B., & De la Fuente, J. (2008). The revised two-factor study process questionnaire (R-SPQ-2F): Exploratory and confirmatory factor analyses at item level. *European Journal of Psychology of Education, 23*(3), 355-372.

García-Berbén, A.B., Pichardo, M.C. & de la Fuente, J. (2007). Relaciones entre preferencias de la enseñanza y enfoques de aprendizaje de los universitarios. *Infancia y Aprendizaje, 30*(4), 537-550, DOI: 10.1174/021037007782334319

de la Fuente J, Fernández-Cabezas M, Cambil M, Vera MM, González-Torres MC and Artuch-Garde R (2017). Linear Relationship between Resilience, Learning Approaches, and Coping Strategies to Predict Achievement in Undergraduate Students. *Front. Psychol. 8*:1039. doi: 10.3389/fpsyg.2017.01039

de la Fuente, J., Peralta-Sánchez, F. J., Martínez-Vicente, J. M., Santos, F. H., Fadda, S., & Gaeta-González, M. L. (2020). Do learning approaches set the stage for emotional well-being in college students? *Sustainability, 12*(17), 6984. https://doi.org/10.3390/su12176984

de la Fuente J, Sander P, Kauffman DF and Yilmaz-Soylu, M (2020). Differential Effects of Self- vs. External-Regulation on Learning Approaches, Academic Achievement, and Satisfaction in Undergraduate Students. *Front. Psychol. 11:*543884. doi: 10.3389/fpsyg.2020.543884

de la Fuente, J. y Zapata, L. (2026). Personal Self-regulation, learning Appraches and Coping Strategies, In Univerity teaching and Learning Processess with Stress. Ed: Independently published (Amazon: Serie Education & Psychology I+D+I).

**2.1.2. Procedural sub-competence**

**· Meta-cognitive Skills: Learning, Study and Note-Taking Strategies**

García- Usero, M. y de la Fuente, J. (coord.) (2001). *Autorregulación del Aprendizaje en el aula*. Sevilla. Junta de Andalucía.

de la Fuente, J., Amate, J., & González-Torres, M. C. (2015). Assessing self-regulated learning and its relation to cognitive performance in early childhood education*. Early Childhood Education,* 13-36.

Pachón-Basallo M, de la Fuente J, González-Torres MC, Martínez-Vicente JM, Peralta-Sánchez FJ and Vera-Martínez MM (2022). Effects of factors of self-regulation vs. factors of external regulation of learning in self-regulated study. *Front. Psychol. 13*:968733. doi: 10.3389/fpsyg.2022.968733

Sánchez, F. J. P., González, H. M., & de la Fuente Arias, J. (2025). Analysis of Self-Regulation and External Regulation of Learning and Emotion Regulation in Academic Achievement of Students in Secondary Education. *Electronic Journal of Research in Educational Psychology, 23* (3), 567-592.

**· Meta-affective Skills: Coping Strategies**

de la Fuente J., Fernández-Cabezas M, Cambil M, Vera MM, González-Torres MC and Artuch-Garde R (2017). Linear Relationship between Resilience, Learning Approaches, and Coping Strategies to Predict Achievement in Undergraduate Students. *Front. Psychol. 8:*1039. doi: 10.3389/fpsyg.2017.01039

de la Fuente, J., Paoloni, P., Kauffman, D., Yilmaz Soylu, M., Sander, P., & Zapata, L. (2020). Big five, self-regulation, and coping strategies as predictors of achievement emotions in undergraduate students. *International journal of environmental research and public health, 17*(10), 3602. https://doi.org/10.3390/ijerph17103602

de la Fuente, J., Mañas, I., Franco, C., Cangas, A. J., & Soriano, E. (2018). Differential effect of level of self-regulation and mindfulness training on coping strategies used by university students. *International journal of environmental research and public health, 15*(10), 2230. doi:10.3390/ijerph15102230

de la Fuente J, Amate J, González-Torres MC, Artuch R, García-Torrecillas JM and Fadda S (2020). Effects of Levels of Self-Regulation and Regulatory Teaching on Strategies for Coping With Academic Stress in Undergraduate Students. *Front. Psychol. 11:*22. doi: 10.3389/fpsyg.2020.00022

de la Fuente, J., Paoloni, P., Kauffman, D., Yilmaz Soylu, M., Sander, P., & Zapata, L. (2020). Big five, self-regulation, and coping strategies as predictors of achievement emotions in undergraduate students. *International journal of environmental research and public health, 17*(10), 3602. https://doi.org/10.3390/ijerph17103602

**· Meta-motivational skills: Meta-motivational study strategies, engagement during study, resilience**

Artuch-Garde R, González-Torres MC, de la Fuente J, Vera MM, Fernández-Cabezas M and López-García M (2017). Relationship between Resilience and Self-regulation: A Study of Spanish Youth at Risk of Social Exclusion. *Front. Psychol. 8*:612. doi: 10.3389/fpsyg.2017.00612

de la Fuente J, Fernández-Cabezas M, Cambil M, Vera MM, González-Torres MC and Artuch-Garde R (2017). Linear Relationship between Resilience, Learning Approaches, and Coping Strategies to Predict Achievement in Undergraduate Students. *Front. Psychol. 8:*1039. doi: 10.3389/fpsyg.2017.01039

de la Fuente J, González-Torres MC, Artuch-Garde R, Vera-Martínez MM, Martínez-Vicente JM and Peralta-Sánchez FJ (2021). Resilience as a Buffering Variable Between the Big Five Components and Factors and Symptoms of Academic Stress at University*. Front. Psychiatry 12:*600240. doi: 10.3389/fpsyt.2021.600240

Artuch-Garde, R., González-Torres, M.C., Martínez-Vicente, J. M., Peralta-Sánchez, F. J., & de la Fuente-Arias, J. (2022). Validation of the child and youth resilience measure-28 (CYRM-28) among Spanish youth. *Heliyon, 8*(6). https://doi.org/10.1016/j.heliyon.2022.e09713

de la Fuente J, Santos FH, Garzón-Umerenkova A, Fadda S, Solinas G and Pignata S (2021) Cross-Sectional Study of Resilience, Positivity and Coping Strategies as Predictors of Engagement-Burnout in Undergraduate Students: Implications for Prevention and Treatment in Mental Well-Being. *Front. Psychiatry 12*:596453. doi: 10.3389/fpsyt.2021.596453

de la Fuente J, Urien B, Luis EO, González-Torres MC, Artuch-Garde R and Balaguer A (2022). The Proactive-Reactive Resilience as a Mediational Variable Between the Character Strength and the Flourishing in Undergraduate Students. Front. Psychol. 13:856558. doi: 10.3389/fpsyg.2022.856558

**· Meta-behavioral skills: Self-regulation strategies during learning and study, long- and short-term self-control, self-control (Self-regulation learning; Self-control long & short time; Self-control)**

de la Fuente, J. (2010). Estrategias metodológicas y de evaluación para promover la competencia para aprender a aprender*. Aula de Innovación Educativa, 192*, 11-14.

de la Fuente, J. y Martínez-Vicente, J.M. (2004). *Competencia para aprender a aprender. Pro & Regula Program.* Málaga: Aljibe.

de la Fuente J, Sander P, Martínez-Vicente JM, Vera M, Garzón A and Fadda S (2017). Combined Effect of Levels in Personal Self-Regulation and Regulatory Teaching on Meta-Cognitive, on Meta-Motivational, and on Academic Achievement Variables in Undergraduate Students. *Front. Psychol. 8:*232. doi: 10.3389/fpsyg.2017.00232

Martínez-Vicente, J. M., & de la Fuente Arias, J. (2004). La autorregulación del aprendizaje a través del Programa Pro&Regula. *Electronic Journal of Research in Education Psychology, 2*(3), 145-156. <https://doi.org/10.25115/ejrep.v2i3.1144>

Umerenkova, A. G., & de la Fuente Arias, J. (2024). Self-regulation assessment: A validation study. *Electronic Journal of Research in Educational Psychology, 22* (2), 347-372.

de la Fuente, J. y Eissa, A. (2025*). International Handbook on Applying Self-Regulated Learning in Different Settings (*vol, I y II). Second Edition. Ed: Independently published (Amazon: Serie Education & Psychology I+D+I).

de la Fuente, J. y Zapata, L. (2026). Personal Self-regulation, Learning Approaches and Coping Strategies, In University teaching and Learning Processess with Stress. Ed: Independently published (Amazon: Serie Education & Psychology I+D+I).

**·Dysfunctional skills: Regulatory fatigue, Executive dysfunction, Emotional dysregulation, Procrastination**

**Regulatory Fatigue**

de la Fuente J, Ubani E, Karagiannopoulou E and Kauffman DF (2025) Effect of Combined Regulatory Behavior Index (CRBI) on regulatory fatigue and behavioral adaptability (classical and reformulated) in the university context. *Front. Psychol. 16:*1533725. doi: 10.3389/fpsyg.2025.1533725

**Executive dys-function**

de la Fuente J, Martínez-Vicente JM, Pachón-Basallo M, Peralta-Sánchez FJ, Vera-Martínez MM and Andrés-Romero MP (2022). Differential Predictive Effect of Self-Regulation Behavior and the Combination of Self- vs. External Regulation Behavior on Executive Dysfunctions and Emotion Regulation Difficulties, in University Students. *Front. Psychol. 13:*876292. doi: 10.3389/fpsyg.2022.876292

de la Fuente J, Fuentes LJ, Santos FH, Pichardo MC and Díaz-Orueta U (2023). Editorial: Executive functions, self-regulation and external-regulation: relations and new evidence. *Front. Psychol. 14:*1335354. doi: 10.3389/fpsyg.2023.1335354

**Procrastination**

Garzón-Umerenkova A, de la Fuente J, Amate J, Paoloni PV, Fadda S and Pérez JF (2018). A Linear Empirical Model of Self-Regulation on Flourishing, Health, Procrastination, and Achievement, Among University Students. *Front. Psychol. 9,*536. doi: 10.3389/fpsyg.2018.00536

Garzón-Umernkova, A., Flores, J. G., & de la Fuente-Arias, J. (2020). Rasgos demográficos, académicos y personales asociados a tres tipos de procrastinación en el alumnado universitario. Bordón. *Revista de pedagogía, 72*(1), 49-65.

de la Fuente J, Sander P, Garzón-Umerenkova A, Vera-Martínez MM, Fadda S and Gaetha ML (2021). Self-Regulation and Regulatory Teaching as Determinants of Academic Behavioral Confidence and Procrastination in Undergraduate Students. *Front. Psychol. 12:*602904. doi: 10.3389/fpsyg.2021.602904

Garzón-Umerenkova, A. G., de la Fuente, J., & Flores, J. G. (2022). Relationship between academic procrastination, well-being, and grades: the mediational role of self-regulation and bad habits. *J. Posit. Psychol. Wellbeing, 6,* 1247-1262.

Garzón-Umerenkova, A., de la Fuente-Arias, J., & Acelas-Russi, L. (2019). Características y dimensiones de la procrastinación académica en estudiantes universitarios colombianos. *Diálogos sobre investigación. Cap. 3.*

**2.1.3. Attitudinal sub-competences**

**· Strengs Psychological**

Villacís, J. L., de la Fuente, J., & Naval, C. (2021). Good character at college: The combined role of second-order character strength factors and phronesis motivation in undergraduate academic outcomes. *International Journal of Environmental Research and Public Health, 18*(16), 8263. https://doi.org/10.3390/ijerph18168263

Villacís, J. L., Naval, C., & de la Fuente, J. (2023). Character strengths, moral motivation and vocational identity in adolescents and young adults: A scoping review. *Current Psychology, 42*(27), 23448-23463.

de la Fuente J, Urien B, Luis EO, González-Torres MC, Artuch-Garde R and Balaguer A (2022). The Proactive-Reactive Resilience as a Mediational Variable Between the Character Strength and the Flourishing in Undergraduate Students. *Front. Psychol. 13:*856558. doi: 10.3389/fpsyg.2022.856558

**· Achievement Emotion: Class, Study, Exam**

de la Fuente J, Martínez-Vicente JM, Peralta-Sánchez FJ, Garzón-Umerenkova A, Vera MM and Paoloni P (2019). Applying the SRL vs. ERL Theory to the Knowledge of Achievement Emotions in Undergraduate University Students. *Front. Psychol. 10:*2070. doi: 10.3389/fpsyg.2019.02070

de la Fuente, J., Lahortiga-Ramos, F., Laspra-Solís, C., Maestro-Martín, C., Alustiza, I., Aubá, E., & Martín-Lanas, R. (2020). A structural equation model of achievement emotions, coping strategies and engagement-burnout in undergraduate students: A possible underlying mechanism in facets of perfectionism. *International journal of environmental research and public health, 17*(6), 2106. <https://doi.org/10.3390/ijerph17062106>

de la Fuente, J., Paoloni, P., Kauffman, D., Yilmaz Soylu, M., Sander, P., & Zapata, L. (2020). Big five, self-regulation, and coping strategies as predictors of achievement emotions in undergraduate students. *International journal of environmental research and public health, 17*(10), 3602. https://doi.org/10.3390/ijerph17103602

de la Fuente J, Kauffman DF and Yilmaz Soylu M (2022) Editorial: Achievement Emotions in University Teaching and Learning, Students' Stress and Well-being. Front. Psychol. 13:910980. doi: 10.3389/fpsyg.2022.910980

**· Test Anxiety**

de la Fuente, J., García-Torrecillas, J. M., & Rodríguez-Vargas, S. (2015). The relationship between coping strategies, test anxiety, and burnout-engagement behavior in university undergraduates. *Coping strategies and health,* 27-44. NY: Nova Science Publishers.

de la Fuente, J., López-García, M., Mariano-Vera, M., Martínez-Vicente, J. M., & Zapata, L. (2017). Personal self-regulation, learning approaches, resilience and test anxiety in psychology students. *Estudios sobre educación, 32*, 9-26.

Amate-Romera, J. A., & de la Fuente, J. (2021). Relationships between test anxiety, self-regulation and strategies for coping with stress, in professional examination candidates. *Anales de Psicología / Annals of Psychology, 37*(2), 276-286. https://doi.org/10.6018/analesps.411131

**· Academic Confidence**

de la Fuente, J., & Sander, P. (2012). Effects of academic confidence and gender on the perception of the teaching-learning process at university. In *Learning Strategies, Expectation and Challenges,* eds E. Maxwell and OA Stephen (New York, NY: Nova Publisher), 93-107.

Sander, P., de la Fuente-Arias, J., Stevenson, K., & Jones, T. (2011). A validation of the academic behavioural confidence scale with spanish psychology students. *Psychology Learning & Teaching, 10*(1), 11-24. <http://dx.doi.org/10.2304/plat.2011.10.1.11>

de la Fuente, J., Sander, P., & Putwain, D. (2013). Relationship between undergraduate student confidence, approach to learning and academic performance: The role of gender. *Revista de Psicodidáctica, 18*(2), 375-393. DOI: 10.1387/RevPsicodidact.7078

Sander, P., & de la Fuente, J. (2020). Undergraduate student gender, personality and academic confidence. *International Journal of Environmental Research and Public Health, 17*(15), 5567. doi:10.3390/ijerph17155567

Sander, P., & de la Fuente, J. (2022). Modelling students’ academic confidence, personality and academic emotions. *Current Psychology, 41*(7), 4329-4340. https://doi.org/10.1007/s12144-020-00957-0

**· Action-emotion style**

de la Fuente, J., & Cardelle-Elawar, M. (2009). Research on action–emotion style and study habits: Effects of individual differences on learning and academic performance of undergraduate students. *Learning and Individual Differences, 19*(4), 567-576. <https://doi.org/10.1016/j.lindif.2009.07.009>

de la Fuente, J., Cardelle-Elawar, M., Sander, P., & Putwain, D. (2013). Action-emotion style, test anxiety and resilience in undergraduate students. In. *Student Learning: Objectives, Opportunities and Outcomes*, 139-149. NY: Nova Publishers Incorporation

de la Fuente, J., Martínez-Vicente, J. M., Salmerón, J. L., Vera, M. M., & Cardelle-Elawar, M. (2016). Action-emotion style, learning approach and coping strategies, in undergraduate university students. *Anales De Psicología/Annals of Psychology, 32*(2), 457-465. <http://dx.doi.org/10.6018/analesps.32.2.197991>

**· Academic perfectionism**

de la Fuente, J., Lahortiga-Ramos, F., Laspra-Solís, C., Maestro-Martín, C., Alustiza, I., Aubá, E., & Martín-Lanas, R. (2020). A structural equation model of achievement emotions, coping strategies and engagement-burnout in undergraduate students: A possible underlying mechanism in facets of perfectionism. *International journal of environmental research and public health, 17*(6), 2106. https://doi.org/10.3390/ijerph17062106

**2.2. Contextual level**

**2.2.1. Academic Situational Stress**

de la Fuente, J., Paoloni, P. V., Vera-Martínez, M. M., & Garzón-Umerenkova, A. (2020). Effect of levels of self-regulation and situational stress on achievement emotions in undergraduate students: class, study and testing*. International journal of environmental research and public health, 17*(12), 4293. https://doi.org/10.3390/ijerph17124293

de la Fuente J, Peralta-Sánchez FJ, Martínez-Vicente JM, Sander P, Garzón-Umerenkova A and Zapata L (2020) Effects of Self-Regulation vs. External-Regulation on the Factors and Symptoms of Academic Stress in Undergraduate Students. *Front. Psychol. 11*:1773. doi: 10.3389/fpsyg.2020.01773

**2.2.2. Regulatory vs. dysregulatory teaching**

de la Fuente-Arias, J., & Justicia, F. J. (2003). Regulación de la enseñanza para la autorregulación del aprendizaje en la Universidad. *Aula abierta*, *82*, 161-172.

de la Fuente-Arias, J., Justicia, F. J., & Berben, A. B. G. (2005). An interactive model of regulated teaching and self-regulated learning. *International Journal of Learning, 12*(7), 121-332.

Justicia, F., de la Fuente, J., Pichardo, M. C., & Berben, A. B. G. (2006). Teaching and evaluation methods preferred by university students. *International journal of learning, 12*(7), 333-340.

de la Fuente-Arias, J., & Justicia, F. J. (2007). El Modelo DIDEPRO® de Regulación de la Enseñanza y del Aprendizaje: avances recientes. *Electronic Journal of Research in Educational Psychology, 5*(3), 535-564.

de la Fuente-Arias, J., & Medialdea, A. M. L. (2007). Modelo de Asesoramiento en I+ D+ i para la mejora del Proceso de Enseñanza-Aprendizaje*. Electronic Journal of Research in Educational Psychology, 5(*3), 879-908. <https://doi.org/10.25115/ejrep.v5i13.1255>

García-Berbén, A.B., Pichardo, M.C. & de la Fuente, J. (2007). Relaciones entre preferencias de la enseñanza y enfoques de aprendizaje de los universitarios. *Infancia y Aprendizaje, 30*(4), 537-550, DOI: 10.1174/021037007782334319

Sánchez-Roda, M. D., de la Fuente, J., & Peralta Sánchez, F. J. (2007). Improving the Teaching-Learning Process through Psychoeducational Advising. *Electronic Journal of Research in Educational Psychology, 13 (*5), 853-878.

de la Fuente-Arias, J., Martínez-Vicente, J. M., Sánchez, F. J. P., & Berbén, A. B. G. (2010). Percepción del proceso de enseñanza-aprendizaje y rendimiento académico en diferentes contextos instruccionales de la Educación Superior. *Psicothema, 22*(4), 806-812.

de la Fuente, J., Sander, P., Justicia, F., Pichardo, M. C., & García-Berbén, A. B. (2010). Validation study of the scale for assessment of the teaching-learning process, student version (ATLP-S). *Electronic Journal of Research in Educational Psychology, 8*(2), 815-840.

de la Fuente, J. (2011). Implications for the DEDEPRO model for interactive analysis of the teaching-learning process in higher education. *Higher education in a state of crisis,* 205-222. NY: Nova Incorporation Publishers.

de la Fuente, J., Cardelle-Elawar, M., Peralta, F. J., Sánchez, M. D., Martínez-Vicente, J. M., & Zapata, L. (2011). Students’ factors affecting undergraduates’ perceptions of their teaching and learning process within ECTS experience. *Frontiers in psychology, 2,* 28. doi: 10.3389/fpsyg.2011.00028

de la Fuente, J., Zapata, L., Martínez-Vicente, J. M., Cardelle-Elawar, M., Sander, P., Justicia, F., ... & García-Belén, A. B. (2012). Regulatory teaching and self-regulated learning in college students: confirmatory validation study of the IATLP scales. *Electronic Journal of Research in Education Psychology, 10*(27), 839-866. <https://doi.org/10.25115/ejrep.v10i27.1511>

de la Fuente, J., García-Berbén, A. B., & Zapata, L. (2013). How regulatory teaching impacts university students' perceptions of the teaching-learning process: The role of teacher training*. Journal for the Study of Education and Development, 36*(3), 375-385. https://doi.org/10.1174/021037013807533016

de la Fuente, J., Justicia, F., Sander, P., & Cardelle-Elawar, M. (2014). Personal self-regulation and regulatory teaching to predict performance and academic confidence: New evidence for the DEDEPRO Model TM. *Electronic Journal of Research in Education Psychology, 12*(34), 597-620. <https://doi.org/10.25115/ejrep.34.14031>

de la Fuente J, Zapata L, Martínez-Vicente JM, Sander P and Cardelle-Elawar M (2015). The role of personal self-regulation and regulatory teaching to predict motivational-affective variables, achievement, and satisfaction: a structural model. *Front. Psychol. 6:*399. doi: 10.3389/fpsyg.2015.00399

de la Fuente, J., Sander, P., Cardelle-Elawar, M., & Pignatta, S. (2016). Effects of level of regulatory teaching on achievement emotions in the learning process; anxiety and coping strategies in Higher Education*. Teaching and learning: Principles, approaches and impact assessment,* 131-150. NY: Nova Publisher Incorp.

de la Fuente, J., Zapata, L., Sander, P. & Putwain, D. (2016). An empirical model of personal self-regulation and teaching regulatory, to predict the process and the product variables. (2014). Revista INFAD De Psicología. *International Journal of Developmental and Educational Psychology, 4*(1), 197-208. https://doi.org/10.17060/ijodaep.2014.n1.v4.603

de la Fuente J, Amate J, González-Torres MC, Artuch R, García-Torrecillas JM and Fadda S (2020). Effects of Levels of Self-Regulation and Regulatory Teaching on Strategies for Coping with Academic Stress in Undergraduate Students. *Front. Psychol. 11*:22. doi: 10.3389/fpsyg.2020.00022

de la Fuente J, Sander P, Garzón-Umerenkova A, Vera-Martínez MM, Fadda S and Gaetha ML (2021). Self-Regulation and Regulatory Teaching as Determinants of Academic Behavioral Confidence and Procrastination in Undergraduate Students. *Front. Psychol. 12:*602904. doi: 10.3389/fpsyg.2021.602904

de la Fuente J, Pachón-Basallo M, Santos FH, Peralta-Sánchez FJ, González-Torres MC, Artuch-Garde R, Paoloni PV and Gaetha ML (2021). How Has the COVID-19 Crisis Affected the Academic Stress of University Students? The Role of Teachers and Students. *Front. Psychol. 12:*626340. doi: 10.3389/fpsyg.2021.626340

de la Fuente, J. (2023). *Protocol for Evaluating the Teaching-Learning Process in the European Space for Higher Education.* Ed: Independently published (Amazon: Serie Education & Psychology I+D+I).

de la Fuente, J. Peralta-Sánchez, FJ y Sánchez-Roda, FJ (2023). *Evaluación y mejora del proceso de enseñanza-aprendizaje en educación secundaria y bachillerato*. Ed: Independently published (Amazon: Serie Education & Psychology I+D+I).

de la Fuente, J., Basallo, M. P., Martínez-Vicente, J. M., Peralta-Sánchez, F. J., López-Andrés, M. P., & García-Torrecillas, J. M. (2025). Evaluation of Competence Achievement and Student Satisfaction in University Teaching and Learning Process*. Electronic Journal of Research in Education Psychology, 23*(66), 299-320.

**3. Product Variables:**

**3.1. Individual variables**

**3.1.1. Psychological Well-being**

López-Madrigal, C., García-Manglano, J., & de la Fuente Arias, J. (2022). A path analysis model of self-vs. educational-context-regulation as combined predictors of well-being in Spanish college students*. International Journal of Environmental Research and Public Health, 19*(16), 10223.

de la Fuente, J. y Martínez-Vicente, JM (2025). *El Índice Experiencia de Bienestar Escolar o Académico (IEBEA) como indicador de la Calidad Educativa.* Ed: Independently published (Amazon: Serie Education & Psychology I+D+I).

de la Fuente-Arias, J., Martínez-Vicente, J. M., Peralta Sánchez, F. J., Pachón-Basallo, M., & Garzón-Umerenkova, A. (2025). Experience Academic Wellbeing Index as Predictor of Psychological Wellbeing and Flourishing. *New Directions for Child and Adolescent Development, 2025*(1), 8862531. https://doi.org/10.1155/cad/8862531

**3.1.2. Flourishing**

Garzón-Umerenkova A, de la Fuente J, Amate J, Paoloni PV, Fadda S and Pérez JF (2018). A Linear Empirical Model of Self-Regulation on Flourishing, Health, Procrastination, and Achievement, Among University Students. *Front. Psychol. 9,*536. doi: 10.3389/fpsyg.2018.00536

de la Fuente J, Santos FH, Garzón-Umerenkova A, Fadda S, Solinas G and Pignata S (2021). Cross-Sectional Study of Resilience, Positivity and Coping Strategies as Predictors of Engagement-Burnout in Undergraduate Students: Implications for Prevention and Treatment in Mental Well-Being. *Front. Psychiatry 12*:596453. doi: 10.3389/fpsyt.2021.596453

**3.1.3. Academic Health**

Garzón-Umerenkova A, de la Fuente J, Amate J, Paoloni PV, Fadda S and Pérez JF (2018). A Linear Empirical Model of Self-Regulation on Flourishing, Health, Procrastination, and Achievement, Among University Students. *Front. Psychol. 9*,536. doi: 10.3389/fpsyg.2018.00536

de la Fuente, J. y Martínez-Vicente, JM (2025). *El Índice Experiencia de Bienestar Escolar o Académico (IEBEA) como indicador de la Calidad Educativa.* Ed: Independently published (Amazon: Serie Education & Psychology I+D+I).

**3.1.4. Academic Stress**

de la Fuente, J., & Amate, J. (2019). Unpleasant past experience as a determinant of cognitive, behavioral and physiological responses to academic stress in professional examination candidates. *Anales de Psicología / Annals of Psychology*, *35*(3), 472-482. <http://dx.doi.org/10.6018/analesps.35.3.323101>

de la Fuente, J. y Zapata, L. (2026). Personal Self-regulation, Learning Approaches and Coping Strategies, In Univerity teaching and Learning Processes with Stress. Ed: Independently published (Amazon: Serie Education & Psychology I+D+I).

**3.1.5. Engagement-burnout**

de la Fuente, J., Zapata, L., Vera, M. M., González-Torres, M. C., & Artuch, R. (2014). Bullying, personal self-regulation, resilience, coping strategies and engagement-burnout: implications for an intervention with university students*. Bullying, prevalence, psychological impact, and strategies intervention,* 91-107. NY: Nova Publishers Incorp.

de la Fuente, J., Lahortiga-Ramos, F., Laspra-Solís, C., Maestro-Martín, C., Alustiza, I., Aubá, E., & Martín-Lanas, R. (2020). A structural equation model of achievement emotions, coping strategies and engagement-burnout in undergraduate students: A possible underlying mechanism in facets of perfectionism. *International journal of environmental research and public health, 17*(6), 2106. <https://doi.org/10.3390/ijerph17062106>

de la Fuente J, Santos FH, Garzón-Umerenkova A, Fadda S, Solinas G and Pignata S (2021). Cross-Sectional Study of Resilience, Positivity and Coping Strategies as Predictors of Engagement-Burnout in Undergraduate Students: Implications for Prevention and Treatment in Mental Well-Being. *Front. Psychiatry 12*:596453. doi: 10.3389/fpsyt.2021.596453

**3.2. Academic Achievement**

Sander, P., Putwain, D., & de la Fuente, J. (2014). Using structural equation modelling to understand predictors of undergraduate students’ academic performance. In *Theory and method in higher education research* (pp. 219-241). Emerald Group Publishing Limited.

Garzón-Umerenkova A, de la Fuente J, Amate J, Paoloni PV, Fadda S and Pérez JF (2018). A Linear Empirical Model of Self-Regulation on Flourishing, Health, Procrastination, and Achievement, Among University Students. *Front. Psychol. 9,*536. doi: 10.3389/fpsyg.2018.00536

de la Fuente J, Sander P, Kauffman DF and Yilmaz Soylu M (2020). Differential Effects of Self- vs. External-Regulation on Learning Approaches, Academic Achievement, and Satisfaction in Undergraduate Students. *Front. Psychol. 11:*543884. doi: 10.3389/fpsyg.2020.543884

de la Fuente, J., Basallo, M. P., Martínez-Vicente, J. M., Peralta-Sánchez, F. J., López-Andrés, M. P., & García-Torrecillas, J. M. (2025). Evaluation of Competence Achievement and Student Satisfaction in University Teaching and Learning Process*. Electronic Journal of Research in Education Psychology, 23*(66), 299-320. https://doi.org/10.25115/ejrep.v23i66.9720

**4. Conclusions; use of the Model and e-Utility**

**4.1. Academic implications: use for research and academic training**

de la Fuente, J., López-Medialdea, M., Zapata, L., Sollinas, G., & Fadda, S. (2015). Improving mental Health trough and online self-assessment and self-help e-Utility in university Students. *Progress in education, 33,* 63-74. NY: Nova Science Publishers.

de la Fuente J, Martínez-Vicente JM, Peralta-Sánchez FJ, González-Torres MC, Artuch R and Garzón-Umerenkova A (2018) Satisfaction With the Self-Assessment of University Students Through e-Coping With Academic Stress Utility ^TM^. *Front. Psychol. 9:*1932. doi: 10.3389/fpsyg.2018.01932

de la Fuente, J., López-Medialdea, M., Zapata, L., Martínez-Vicente, J. M., Vera, M. M., Solinas, G., & Fadda, S. (2014). Competency to Study and Learn in Stressful Contexts: Fundamentals of the e-Coping with Academic Stress Utility ^TM^. *Electronic Journal of Research in Educational Psychology, 12*(3), 717-746.

de la Fuente J (2021). A Path Analysis Model of Protection and Risk Factors for University Academic Stress: Analysis and Psychoeducational Implications for the COVID-19 Emergency. *Front. Psychol. 12:*562372. doi: 10.3389/fpsyg.2021.562372

de la Fuente J, Kauffman DF, Dempsy MS and Kauffman Y (2021). Analysis and Psychoeducational Implications of the Behavior Factor During the COVID-19 Emergency. *Front. Psychol. 12:*613881. doi: 10.3389/fpsyg.2021.613881

de la Fuente, J. (2025). Concepciones Académicas y Profesionales para la Promoción del Bienestar en Contextos Educativos, desde la Psicología Educativa. *Informació Psicològica, 128*, 3-12. <https://doi.org/10.70478/ipsic.2008>

**4.2. Professional implications: use for professional simulations with case studies**

de la Fuente, J., Amate-Romera, J., & Sander, P. (2018). Relaciones entre estrategias cognitivas, estrategias motivacionales y estrés académico en universitarios opositores. *Electronic Journal of Research in Educational Psychology, 16*(2), 345-365.

de la Fuente, J. (2025). Concepciones Académicas y Profesionales para la Promoción del Bienestar en Contextos Educativos, desde la Psicología Educativa. *Informació Psicològica, 128*, 3-12. <https://doi.org/10.70478/ipsic.2008>

de la Fuente, J., y Martínez Vicente, J. M. (2025). El “índice de experiencia de bienestar escolar y académico” como estrategia de evaluación e intervención psicoeducativa y correlato del bienestar psicológico. *Informació Psicològica, 128,* 13-23. <https://doi.org/10.70478/ipsic.2010>

de la Fuente, J., Martínez-Vicente, J. M., y Vera Martínez, M. M. (2025). La necesidad de implementar estrategias para aportar evidencia científico-profesional desde los centros u organizaciones educativas: las unidades o departamentos de I+D+I. *Informació Psicològica, 128,* 25-32. https://doi.org/10.70478/ipsic.2011

de la Fuente, J., y Pachón-Basallo, M. (2025). Experiencias de formación preventiva (primaria y secundaria) con familias y alumnado de psicología, como futuros profesionales. *Informació Psicològica, 128*, 33-42. https:/􀀒doi.org/10.70478/ipsic.2012
